# Effects of Feather-Pecking Phenotype on Physiological and Neurobiological Characteristics and Gut Microbiota Profile of Goslings

**DOI:** 10.3390/ani15142122

**Published:** 2025-07-17

**Authors:** Mingfeng Wang, Yujiao Guo, Zhengfeng Cao, Qi Xu, Guohong Chen, Yang Chen

**Affiliations:** 1 College of Animal Science and Technology, Yangzhou University, Yangzhou 225009, China; 18361310771@163.com (M.W.); yujiaoguo11@163.com (Y.G.); caozhengfeng@yzu.edu.cn (Z.C.); xuqi@yzu.edu.cn (Q.X.); ghchen2019@yzu.edu.cn (G.C.); 2 Joint International Research Laboratory of Agriculture and Agri-Product Safety, The Ministry of Education of China, Yangzhou University, Yangzhou 225009, China; 3 Key Laboratory for Evaluation and Utilization of Poultry Genetic Resources of Ministry of Agriculture and Rural Affairs, Yangzhou University, Yangzhou 225009, China

**Keywords:** feather pecking, gosling, 5-HT, microbiota–gut–brain axis

## Abstract

FP is a detrimental behavior for chickens, ducks, and geese associated with numerous physiological and neurobiological characteristics, which have been identified in many species as regulated by the gut microbiota. However, it is unknown whether and how gut microbiota influences FP by regulating neurotransmitter systems in geese. This study aimed to investigate the phenotypic correlation between feather pecking and changes in physiological, neurobiological, and gut microbiota profiles in gosling. Three behavioral phenotypes were observed in goslings, including severe feather peckers (SFPs), victims of SFPs, and non-peckers (NFPs). The significantly lower feather scores and body weights were observed in victims compared to both SFPs and NFPs (*p* < 0.05). Regarding the physiological phenotype, victims had higher dopamine (DA) levels than NFPs, and SFPs had lower 5-hydroxytryptamine (5-HT) in the serum than NFPs (*p* < 0.001), with intermediate 5-HT levels in victims. Victims had lower glutathione peroxidase (GSH-Px) compared to SFPs and NFPs (*p* < 0.05). Moreover, higher mRNA expression levels of HTR1A, SLC6A4, and TPH2 in the 5-HT metabolic pathway were detected in NFPs than those in SFPs and victims (*p* < 0.05). In addition, regarding gut microbiota measured by 16S rRNA sequencing, SFPs had lower diversity and comparable cecal microbiota compared to victims and NFPs. *Proteobacteria*, *Verrucomicrobia*, *Ruminococcus* spp., and *Bilophila* spp. were enriched in SFPs, while *Bacteroides* and *Parabacteroides* were enriched in NFPs. From the predicted bacterial functional genes, the cAMP signaling pathway, cGMP–PKG signaling pathway, and pyruvate metabolism were activated in SFPs. The correlation analysis revealed that the genera *Bacteroides* spp. were associated with differences in 5-HT metabolism between the SFPs and NFPs. In summary, differences in the cecal microbiota profile and 5-HT metabolism drive FP phenotypes, which could be associated with the reduced gut abundance of the genera *Bacteroides* spp.

## Simple Summary

In summary, our study reveals distinct feather-pecking (FP) phenotypes in goslings through integrated behavioral, physiological, and microbial analyses. Feather-pecked goslings exhibited reduced feather quality, impaired growth performance, and compromised antioxidant capacity. Notably, severe feather peckers demonstrated long-term stress and immunosuppression, characterized by decreased serum 5-HT levels and altered cecal microbiota composition, particularly increased *Bacteroides* spp. with elevated *Proteobacteria* spp. and *Bilophila* spp. These findings highlight the critical roles of gut microbial dysbiosis and 5-HT metabolic dysfunction in FP phenotypic determination.

## 1. Introduction

Feather pecking (FP) has consistently been a severe issue in poultry production management; to date, no efficient prevention and control measures have been established [1,2]. The occurrence of feather-pecking behavior not only results in a decline in animal welfare but also causes significant economic losses [3]. FP can be categorized into gentle feather pecking (GFP) and severe feather pecking (SFP) [4,5]. More importantly, SFP can be learned and transmitted within the group [6,7]. Severe pecking is also the main target trait in current pecking studies and is mostly associated with feeding and foraging behavior, usually occurring under conditions of environmental stress that are difficult to cope with [2,5]. Consequently, FP is an important behavioral problem in poultry production systems that urgently requires resolution.

FP is a multifaceted problem influenced by genetic background, early life history, and environmental factors [5,7]. It may arise from both endogenous factors, such as genetic and physiological factors, and environmental factors, including feeding, density, and housing conditions. The occurrence of feather-pecking behavior is closely associated with oxidative stress and dopaminergic system. Social stress and aggressive behavior can exacerbate dopaminergic system activity and reactive oxygen species (ROS) production, leading to cellular oxidative stress [8]. When ROS are overproduced and not promptly eliminated, cellular function and structure are impaired, further exacerbating aggressive behavior [9]. Several neurotransmitters, including serotonin (5-hydroxytryptamine, 5-HT) and dopamine (DA), have been shown to influence feather-pecking behavior [10,11]. In vertebrates, neural networks and stimulus responses are governed by the brain’s neural network and the secretion of hormones such as 5-HT, DA, epinephrine (E), and corticosterone (CORT) [12,13]. Multiple hormones collectively regulate the pathological physiological states and behaviors of animals [14,15,16]. Low activity of the dopaminergic system in specific brain regions has been linked to the development of FP [17]. Furthermore, the serotonergic system plays a critical role in modulating FP. Deficiencies or excesses in the central serotonergic system can predispose poultry to FP, and individuals with a high propensity for FP typically exhibit lower 5-HT turnover rates during early life stages but higher brain 5-HT turnover rates in adulthood [18]. Adrenocortical activity has been monitored in high- and low-pecking chicks during the first 8 weeks of life, and DA and 5-HT levels in the brains of high-feather-pecking and low-feather-pecking chicks at 28 days of age have been studied [19]. The findings revealed that high-pecking chicks exhibited lower plasma CORT levels and reduced turnover rates of 5-HT and polydopamine. Similarly, the serum levels of tryptophan and 5-HT in high-pecking chickens were lower than those in low-pecking chickens [20]. These observations underscore the intrinsic relationship between FP in poultry and the influence of the central neurotransmitter system, particularly the 5-HT/DA axis.

Recent studies have underscored the pivotal role of gut microbiota in modulating brain function and its profound impact on psychological and emotional stability via the gut–brain axis [21,22,23,24,25,26]. These findings have been complemented by emerging insights into the underlying mechanisms of this axis, particularly focusing on the microbiota’s regulatory effects on key neurotransmitter systems, such as the 5-HT and DA pathways in the central nervous system (CNS), and their immunomodulatory functions [27,28,29]. Münger proposed that the microbiota–gut–brain axis significantly influences host social behavior, emphasizing a strong correlation between gut microbiota composition and social behavioral patterns [30]. The pecking chickens exhibited higher abundances of *Gemmiger* and *Bacteroides*, while *Roseburia*, *Ruminococcus*, *Anaerostipes*, and *Methanobrevibacter* were significantly reduced [31]. Notably, FP can escalate to cannibalistic pecking behavior, which is further reinforced through the gut–brain reward system, mediated by the central 5-HT and DA pathways, leading to the consumption and removal of the victim’s flesh [32,33]. These findings were further supported by conducting a comparative analysis of microbial diversity, gut microbial metabolites, and inflammatory responses between high-feather-pecking and low-feather-pecking laying hens [20]. Their findings revealed that HFP hens exhibited significantly reduced amounts of Firmicutes and Lactobacillus. This provides further evidence that gut microbiota composition is associated with the modulation of behavioral traits and associated physiological responses.

In this study, we investigated the effects of the feather-pecking phenotype on physiological and neurobiological characteristics and gut microbiota profiles in goslings. Specifically, we compared three groups: SFPs, victims, and NFPs. Systemic antioxidant capacity (SOD, MDA, and GSH-Px), stress-related physiological markers (E and CORT), and key neurotransmitter levels (DA and 5-HT) were measured. The gene expression profiles of relevant targets were quantified using RT-qPCR. Additionally, 16S rRNA sequencing was performed to characterize gut microbiota composition and diversity, followed by correlation analysis to explore relationships between microbial profiles and feather-pecking behavior.

## 2. Materials and Methods

### 2.1. Experimental Design and Feeding Management

Seventy-eight Yangzhou goslings were raised in the brooding room of Tiange Industrial Co., Ltd., Yangzhou, China. The experimental procedures were approved by the Ethics Committee on Animal Experiments of Yangzhou University (permit number: YZUDWSY, Government of Jiangsu Province, China). The average temperature was maintained at approximately 28 °C, and the average humidity was controlled at around 65%. To guarantee the even growth of young goslings, 24 h illumination was supplied for those aged 0 to 7 days. Once they reached 8 days old, a gradual shift was made from 24 h lighting to relying solely on natural light. The feeding method was net-bed feeding, equipped with waterers and feeders, and the goslings were provided with meat gosling compound feed produced by Yangzhou Changhong Feed Co., Ltd., Yangzhou, China. Feeding was conducted once at each of the following three time points: 8:00, 14:00, and 20:00, totaling three times a day. The composition and nutrient levels of the goslings’ diet were referenced from previous feeding experiments [34]. Immunization schedules and other husbandry management practices were all implemented following the standard routines for meat goose rearing. Each gosling wore a digitized leg ring on its left leg for easy identification and tracking. The specific feeding conditions were consistent with previous studies [34].

Starting from the third day of age, the feeding patterns and behaviors of the geese were monitored. Data collection spanned from day 3 to day 10, with observations conducted daily between 9:00 and 11:00 and 15:00 and 17:00. For the specific observation methods, you can refer to our previous research [34]. Based on preliminary behavioral observations, the goslings were classified into three categories: severe feather peckers (SFPs), victims of SFP (victims), and non-peckers (NFPs). The specific behavioral discrimination criteria are shown in Table 1, adapted from Kops et al. [3] and Zepp et al. [7]. Once the selected goslings were identified, they were marked with a color on the head (red, purple, and blue, differentiating between different animal classes), and 10 goslings were selected from each group.

### 2.2. Body Weight, Feather Scoring, and Sample Collection

At the end of the behavioral observations (11 days), the selected 30 goslings were weighed and scored for feather coverage according to the feather scoring criteria, which were adapted from Gilani [35] (Table 2). In addition, the blood was collected in a non-anticoagulant vacuum tube. Blood samples were centrifuged at 3000 rpm for 15 min, and separated serum was stored at −20 °C for analysis. Immediately after euthanasia using an overdose of sodium pentobarbital (1.5 mL/kg), the gizzard, cecum, cecal contents, adrenal glands, and brain were carefully excised under aseptic conditions. The gizzard underwent anatomical analysis, the adrenal glands were weighed, and the length of the cecum was measured. The intact brain and cecal contents were immediately frozen using liquid nitrogen and stored at −80 °C for subsequent experimental analysis. The adrenal index was calculated as the ratio of adrenal gland weight to body weight individually.

### 2.3. Analysis of Serum Biochemical Indicators by ELISA

Total superoxide dismutase (T-SOD), malondialdehyde (MDA), glutathione peroxidase (GSH-Px), epinephrine (E), corticosterone (CORT), 5-Hydroxytryptamine (5-HT), and dopamine (DA) were determined using an ELISA kit (Wuhan Eliot Bioscience and Technology Co., Ltd., Wuhan, China) according to the manufacturer’s instructions. The range of the assay and the sensitivity of the assay were as follows: T-SOD (0.2–14.4 U/mL, 0.2 U/mL), MDA (2.92–40 μmol/L, 1.13 μmol/L), GPH-Px (12.65–387 U, 12.65 U), E (18.75 pg/mL, 31.25–2000 pg/mL), CORT (1.89 ng/mL, 3.13–200 ng/mL), 5-HT (9.38 ng/mL, 115.63–1000 ng/mL), and DA (18.75 pg/mL, 31.25–2000 pg/mL). Optical density (OD) values were measured using a microplate reader (Thermo ScientificTM, Waltham, MA, USA), and concentrations in samples were also determined by comparison with a standard curve.

### 2.4. 16S rRNA Gene Sequencing and Bioinformatics Analysis of Cecal Microbiota

Cecal content samples (10 per group: SFPs, victims, and NFPs) were collected and microbial genomic DNA extracted, with integrity verified via 1% agarose gel electrophoresis. The V3–V4 hypervariable regions of the 16S rRNA gene were amplified using the primers 338F (5′-ACTCCTACGGGAGGCAGCAG-3′) and 806R (5′-GGACTACHVGGGTWTCTAAT-3′) in an ABI GeneAmp 9700 (Thermo ScientificTM, Waltham, MA, USA) thermocycler. PCR products were purified with the AxyPrep DNA Gel Extraction Kit (Axygen Biosciences, Union City, CA, USA) and quantified using the QuantiFluor™-ST system (Promega, Madison, WI, USA) per the manufacturer’s instructions. Purified amplicons were sent to Majorbio (Shanghai, China) for Illumina MiSeq sequencing. Sequence data were analyzed on the Majorbio Cloud platforms, including α/β-diversity calculations, principal coordinate analysis (PCoA), and linear discriminant analysis effect size (LEfSe) to identify microbial biomarkers, enabling the characterization of community composition and functional differences across groups.

### 2.5. Quantitative Real-Time PCR (RT-qPCR)

The total RNA was isolated from the brain and caeca using commercial extraction kits procured from TIANGEN Biotech (Beijing, China). The mRNA levels of relevant genes were measured using a real-time quantitative PCR master mix (Vazyme, Q711-02, Nanjing, China) on an ABI 7500 system (Applied Biosystems, located in Foster City, CA, USA) using the primers (Table 3). The relative abundance of the indicated mRNAs normalized to GAPDH in each sample is shown. All data were processed using the 2^−ΔΔCT^ method.

### 2.6. Statistical Analyses

The analyses of statistics were done via SPSS software (IBM SPSS, 22, Armonk, NY, USA). The K–S (Kolmogorov–Smirnov) test examined all data for normal distribution. The data that followed a normal distribution were subjected to a one-way ANOVA, followed by Duncan’s multiple-range test to determine whether significant differences existed between the groups; the results were expressed as the means ± standard deviation. *p* < 0.05 indicates a significant difference. Pearson’s method was used to conduct a correlation analysis between relevant factors such as 5-HT and the gut microbial community. The Mann–Whitney U test was used for the predictive analysis of gut microbiota functions.

## 3. Results

### 3.1. Comparative Analysis of Feather Condition, Growth Performance, Gizzard Contents, and Cecal Morphology

**Figure 1:**
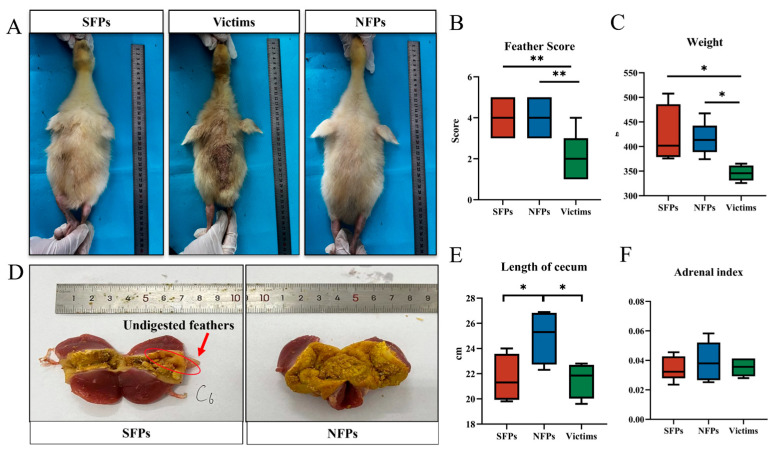
The physical characteristics of the gosling classification. N = 30. (**A**) Three categories of FP goslings: severe feather peckers (SFPs), victims of SFP (victims), and non-peckers (NFPs). (**B**) Feather scores. (**C**) Body weight. (**D**) Feather residue from gizzard contents. (**E**) Length of the cecum. (**F**) Adrenal index. * *p* < 0.05, ** *p* < 0.01.

As illustrated in Figure 1A,B, the feather scores of the victim group were found to be significantly lower than those of the SFP and NFP groups (*p* < 0.01). Similarly, a significant difference in body weight was observed among the groups, with the victim group exhibiting a notably lower body weight compared to both the SFP and NFP groups (*p* < 0.05, Figure 1C).

The post-mortem examination revealed undigested feather remnants in the gizzards of the goslings (Figure 1D), which was likely associated with the observed pecking behavior and feather consumption among the goslings. The gizzard, which primarily functions to grind feed without secreting digestive juices, may contribute to the accumulation of undigested feather material. In terms of cecal length, the NFP group demonstrated a significantly longer cecum compared to the SFP and victim groups (Figure 1E). There was no difference in the adrenal index among the three groups (*p* > 0.05, Figure 1F). Collectively, the victim group exhibited stunted growth, as evidenced by the diminished body weight and inferior feather scores, suggesting impaired growth and development.

### 3.2. Comparison of Serum Antioxidant Capacity and Physiological Stress

**Figure 2:**
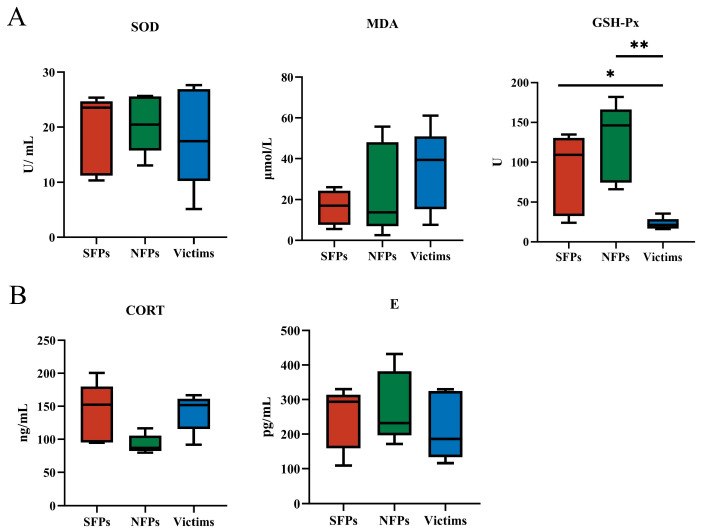
Detection of indicators of serum antioxidant capacity and physiological stress. (**A**) Comparison of serum antioxidant indices of MDA, SOD, and GSH-Px. (**B**) Comparison of hormones E and CORT in goslings. * *p* < 0.05, ** *p* < 0.01.

As depicted in Figure 2A, the victim group exhibited a significant reduction in GSH-Px levels compared to both the SFP and NFP groups (*p* < 0.05). In contrast, no significant differences were observed in MDA or SOD levels across the three groups (*p* > 0.05). The decline in GSH-Px levels within the victim group may indicate a reduced antioxidant capacity, which could potentially impair immune cell function and overall immune status.

As shown in Figure 2B, there were no significant differences in E or CORT levels among the SFP, victim, and NFP groups (*p* > 0.05), suggesting no notable variation in physiological stress markers across the experimental groups, which is consistent with the adrenal index.

### 3.3. Comparison of Serum 5-HT and DA Levels

**Figure 3:**
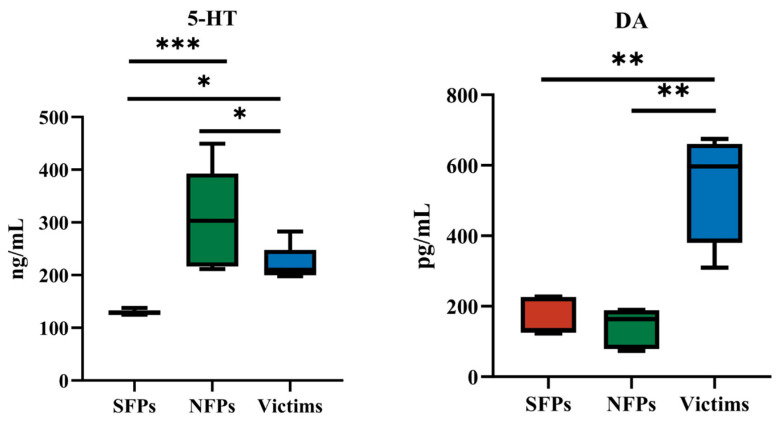
Detection of 5-HT and DA in serum. * *p* < 0.05, ** *p* < 0.01, *** *p* < 0.001.

The serum 5-HT and DA concentrations of SFP, victim, and NFP goslings are listed in Figure 3. The 5-HT concentration of SFPs is significantly down-regulated compared with that of NFPs (*p* < 0.001), suggesting that 5-HT metabolism affects feather-pecking behavior. The victim group of goslings showed significantly higher concentrations of DA levels than those of SFPs and NFPs (*p* < 0.01), indicating that DA metabolism impacts the physiological state of pecked goslings. In summary, the above data indicated that the 5-HT metabolism of SFPs was inhibited, resulting in the reduction of its 5-HT level in SFPs. Central 5-HT could initiate FP and feather eating, and DA could further reinforce this behavior. This indicates that serotonin plays a regulatory role in feather-pecking behavior. Therefore, our research will further focus on the aspect of serotonin.

### 3.4. Comparison of 5-HT Metabolism-Related Gene Expression Differences

**Figure 4:**
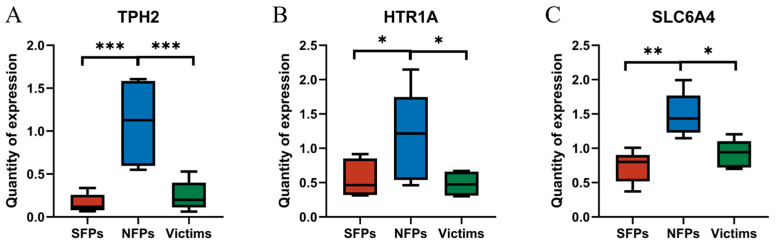
Differential expression of 5-HT metabolism-related genes ((**A**) TPH2, (**B**) HTR1A, and (**C**) SLC6A4). * *p* < 0.05, ** *p* < 0.01, *** *p* < 0.001.

In order to analyze the changes in the metabolism of 5-HT caused by feather pecking, we further employed the RT-qPCR method to detect the expression levels of related genes in the brain, such as HTR1A, SLC6A4, and TPH2 (TPH2 determines the basal production of serotonin in the brain; SLC6A4 controls the duration of serotonin’s effect in the synaptic cleft; and HTR1A maintains a negative feedback balance between serotonin synthesis and release). The expression of HTR1A was significantly higher in the NFP group than in the SFP and victim groups (*p* < 0.05) (Figure 4). Correspondingly, the expression of the SLC6A4 gene was also significantly upregulated in the NFP group in the brain (*p* < 0.05). The gene expression of TPH2 was significantly higher in the NFP group than in the SFP and victim groups (*p* < 0.05).

### 3.5. Diversity of Gut Microbiota in Goslings with Different Pecking Phenotypes

**Figure 5:**
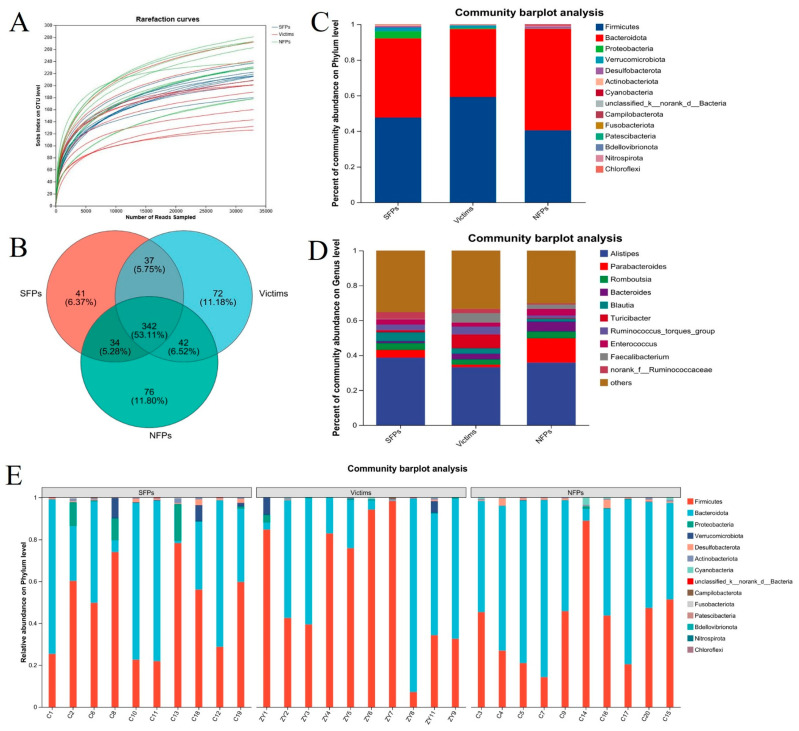
16S rRNA analysis of the gut microbiome of three groups of goslings. (**A**) Sparse curve of intestinal flora. (**B**) Venn diagrams were used to represent the operational taxonomic units (OTUs) observed in three groups of goslings. (**C**,**D**) Estimates of abundance indices at the phylum and genus level for three groups. (**E**) Estimates of abundance indices at the phylum level for three groups of goslings.

As presented in Figure 5A, 16S rRNA sequencing analysis was conducted on the cecal contents of SFP, victim, and NFP goslings. Most rarefaction curves reached a saturated plateau (Figure 5B), suggesting that the sequencing depth was adequate to capture the entire bacterial diversity. A total of 644 operational taxonomic units (OTUs) were identified across all samples. Among these, 41, 72, and 76 OTUs were unique to the SFP, victim, and NFP groups, respectively, and 342 OTUs were shared among the three groups.

Regarding the species-composition analysis, at the phylum level (Figure 5C,E), differences in bacterial abundance were observed. In the SFP group, the cecal flora was mainly composed of *Firmicutes* (47.66%) and *Bacteroidota* (44.38%), with *Proteobacteria* being less abundant (4.09%). The victim group had a similar profile, dominated by *Firmicutes* (59.20%) and *Bacteroidota* (38.14%), while *Verrucomicrobiota* was present in relatively low amounts (1.44%). In contrast, in the NFP group, *Bacteroidota* was the most prevalent phylum (56.91%), followed by *Firmicutes* (40.48%), and *Desulfobacterota* was in low amounts (1.26%).

At the genus level (Figure 5D), *Alistipes* was the dominant genus in the SFP group, accounting for 38.7%, while *Blautia* (5.05%) and *Parabacteroides* (4.37%) were present in lower proportions. In the victim group, *Alistipes* also dominated (33.26%), with *Turicibacter* being less abundant (7.92%). In the NFP group, *Alistipes* was the most abundant genus (35.86%), followed by *Parabacteroides* (13.89%), *Bacteroides* (5.68%), and *Romboutsia* (3.87%) in lower amounts.

**Figure 6:**
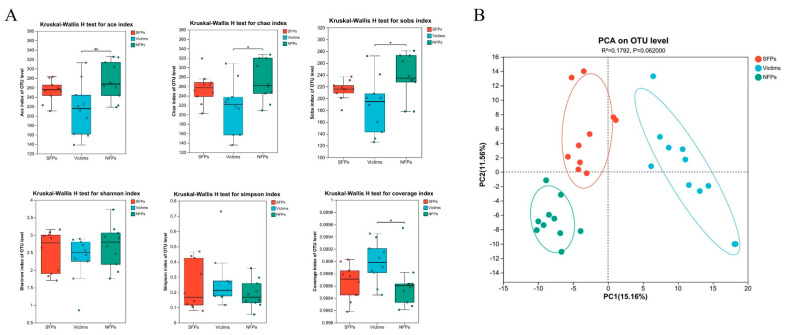
Diversity analysis of gut microbiota in gosling. (**A**) Gut microbiota alpha diversity index in goslings of SFP, victim, and NFP groups. (**B**) The principal coordinates (PCA). * *p* < 0.05, ** *p* < 0.01.

Inter-group variations in alpha diversity indices were meticulously examined, as illustrated in Figure 6A. Highly significant differences were noted between the victim and NFP groups in both the Ace index (*p* < 0.01) and the Chao index (*p* < 0.01), and a less significant difference in the Chao index was also detected (*p* < 0.05). No significant differences were found among the three groups in terms of the Shannon and Simpson indices. Nevertheless, significant differences were identified between the victim and NFP groups for the coverage index and the Sobs index (*p* < 0.05).

For beta diversity analysis, the unweighted UniFrac distance metrics were employed, and the PCA and ANOSIM methods were used to assess the similarity of cecal microbiota among the groups. PCA results showed significant separation among the SFP, victim, and NFP groups (Figure 6B).

### 3.6. Linear Discriminant Analysis and Effect Size Analysis

**Figure 7:**
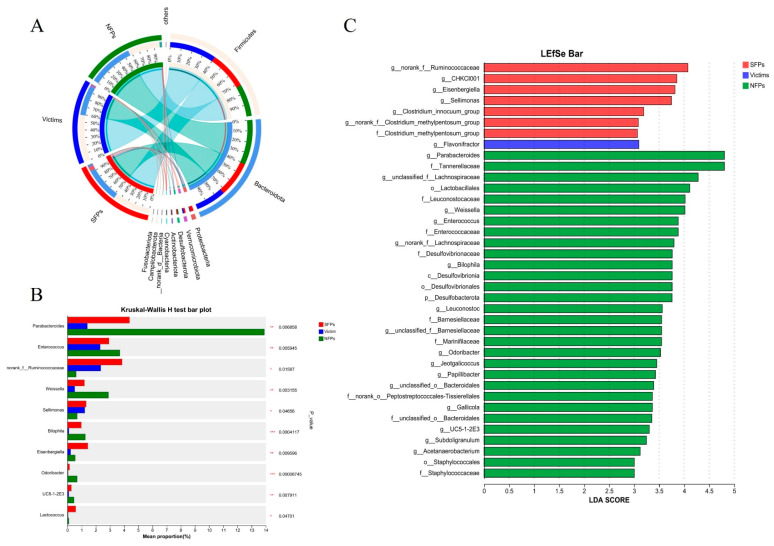
An analysis of the linear discriminant analysis effect size was performed on the gut microbiota of goslings. (**A**) Circos sample species relationship map demonstrating the distribution of microbial species present in the microflora of three groups of goslings. (**B**) Bacterial composition comparisons among the groups on phylum level (Kruskal–Wallis H test). (**C**) LDA scores (LDA > 3) of taxonomic units showing differences in the abundance of SFPs, victims, and NFPs. Red, blue, and green bars correspond to taxonomic units enriched in SFPs, victims, and NFPs, respectively. * *p* < 0.05, ** *p* < 0.01, *** *p* < 0.001.

The ternary analysis demonstrates the community composition and relative abundance of dominant species across the three distinct groups. Species-sample relationships are presented in the form of a boxplot (Figure 7A). Notably, at the genus level (Figure 7B), *parabacteroides*, *Enterococcus*, *Eisenbergiella*, *weissella*, and *UC5-1-2E3* showed highly significant differences among the three groups of SFPs, victims, and NFPs (*p* < 0.01); *Norank_f_Ruminococcaceae*, *Sellimonas,* and *Lactococcus* showed significant difference among the three groups (*p* < 0.05); and *Bilophila* and *Odoribacter* showed highly significant difference among the three groups (*p* < 0.001). To further identify and distinguish the cecum microbiota, we performed LEfSe analysis (Figure 7C). In the SFP group, *g_norank_f_Ruminococcaceae*, *g_CHKCI001*, *g_Eisenbergiella*, and *g_Sellimonas* showed significant differences; in the victim group, *g_Flavonifractor* showed significant differences; in the NFP group, *g_parabacteroides*, *f_tannerellaceae*, *o_lactobacillales*, *f_leuconostocaceac*, *g_weissella*, *g_enterococcus*, and *f_enterococcaceae* presented significant differences.

### 3.7. Integration of Random Forest Analysis and PICRUSt2-Based Functional Prediction

**Figure 8:**
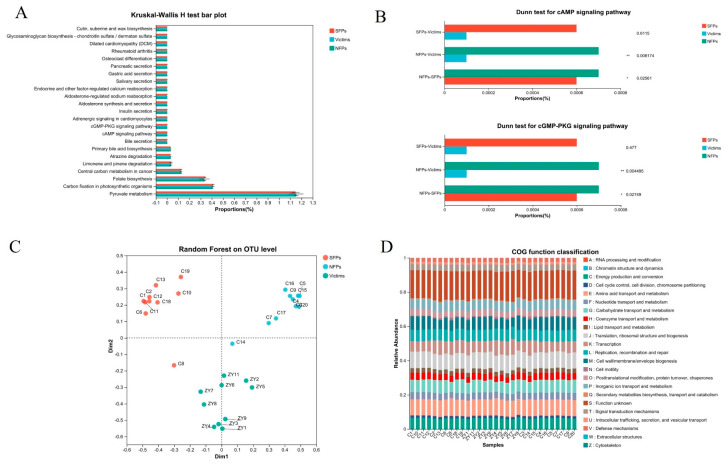
Integration of random forest analysis and PICRUSt2-based functional prediction. (**A**,**B**) PICRUSt2 predicts functional information about microbial communities in environmental samples. (**C**) Random forest analysis. (**D**) Bar graph of COG functional classification statistics. * *p* < 0.05, ** *p* < 0.01.

To infer the functional potential of microbial communities, we utilized PICRUSt2 to characterize the functional profiles of the gut microbiota in goslings. The analysis revealed a significant enrichment of the cAMP signaling pathway, cGMP–PKG signaling pathway, and pyruvate metabolism, as shown in Figure 8A,B. Notably, the cAMP signaling pathway activates protein kinase A (PKA), which modulates the release of neurotransmitters like DA and 5-HT, thus playing a crucial part in the regulation of emotions and behaviors. Moreover, stress or stressful situations can activate these signaling pathways, consequently influencing neuronal metabolism and activity and potentially giving rise to the abnormal behaviors witnessed in goslings. Random forest analysis further revealed distinct clustering patterns among the sample groups, highlighting pronounced inter-group variability (Figure 8C). Additionally, bar charts were employed to illustrate the proportion of COG functional categories across different samples, as displayed in Figure 8D.

### 3.8. Association Analysis of the Gut Microbiota–Antioxidant Indicators–Hormones Axis

**Figure 9:**
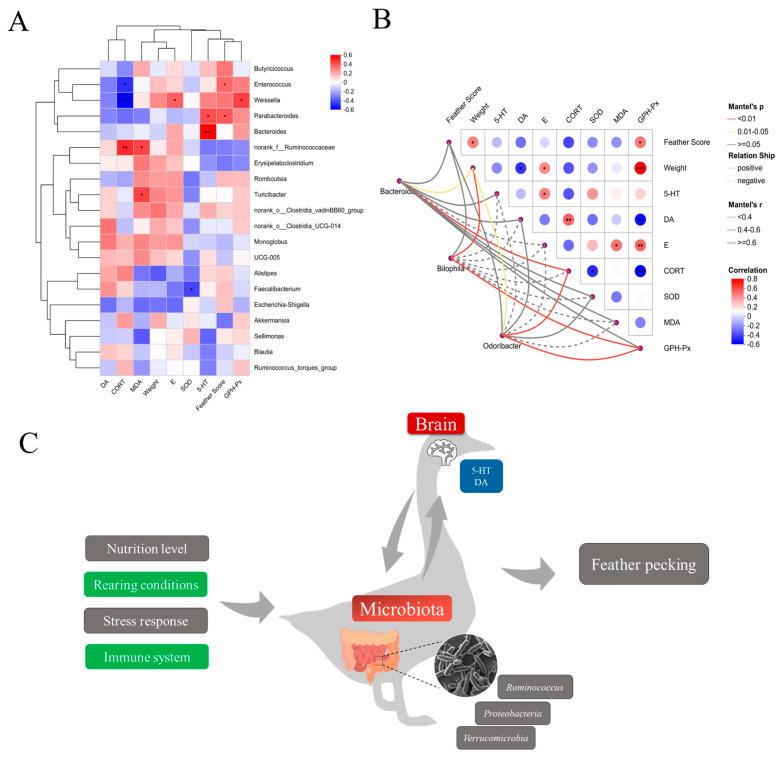
Correlation between gut flora genus, feather score, physiological indices, and antioxidant capacity. (**A**) Spearman correlation coefficients of the correlation between gut flora genus, feather score, physiological indices, and antioxidant capacity with different bacterial genera. (**B**) Correlation between feather score, physiological indices, and antioxidant capacity with *Bacteroides, Bilophila*, and *Odoribacter*. The heatmap shows statistically significant correlation values (r). Red squares indicate significant positive correlation (0 < r ≤ 1), white squares indicate no correlation (r = 0), and blue squares indicate significant negative correlation (1 ≤ r < 0); darker colors indicate greater correlation. * *p* < 0.05, ** *p* < 0.01, *** *p* < 0.001. (**C**) Schematic diagram of the gut microbiota–antioxidant indicators–hormone axis.

To investigate potential associations among FP behavior, physiological alterations, and intestinal microbiota composition, Spearman correlation analyses were performed between the top 30 differentially abundant microbial taxa and feather scores, serum antioxidant capacity, and hormone levels across three groups (Figure 9A). The analyses revealed that the *Bacteroides* had a significant positive correlation with the 5-HT correlation; *Bilophila* spp. had a significant positive correlation with GSH-Px, weight, and feather score; and in addition, *Odoribacter* spp., which belongs to the phylum Bacteroidetes, also showed a significant positive correlation with 5-HT. Among the factors, GSH-Px showed a significant positive correlation with weight (Figure 9B).

The findings reveal that the physiological system, immune system, and microbial community composition are closely associated with feather-pecking behavior. Furthermore, the cecal microbiota may modulate this behavior through interactions between the central nervous system and the gut microbiota, as illustrated in Figure 9C.

## 4. Discussion

This study aims to elucidate the regulatory mechanisms underlying feather-pecking behavior in goslings. Through feather scoring assessments in goslings, we observed that NFPs exhibited higher feather scores, while victims displayed significantly lower scores, consistent with previous findings [7,20,35]. Victims experienced more frequent feather-pecking behaviors, resulting in greater feather damage. Notably, feather pecking led to a significant reduction in the body weight of victims, aligning with the results reported by Tahamtani et al. [36]. These findings suggest that feather pecking disrupts the stable development of goslings and negatively impacts their early rearing performance.

In investigating the role of the antioxidative system in feather-pecking behavior, our study revealed that victims exhibited reduced levels of GSH-Px, consistent with findings by Surai [37]. GSH-Px, a critical antioxidant enzyme ubiquitous in organisms, plays a vital role in maintaining redox homeostasis. Disruption of the redox balance and the onset of oxidative stress can influence animal behavior. A decline in GSH-Px activity impairs the organism’s ability to effectively eliminate peroxides, leading to elevated intracellular oxidative stress levels [38,39]. This oxidative stress may disrupt neurotransmitter metabolism and neural function, particularly interfering with 5-HT metabolism, which can result in heightened anxiety and irritability, thereby increasing the likelihood of feather-pecking behavior. Additionally, the skin damage caused by feather pecking elevates the risk of infection, further exacerbating disruptions in the body’s redox state and GSH-Px activity [40,41].

In the physiological and nervous systems, current research predominantly focuses on the 5-HT system. Studies have demonstrated that chickens with a HFP genotype exhibit lower peripheral 5-HT levels [19,20,42]. Our findings corroborate these observations. Specifically, SFP goslings exhibited significantly lower serum 5-HT levels compared to normal goslings. Low 5-HT levels have been associated with heightened susceptibility to anxiety and depressive states, thereby increasing the likelihood of feather-pecking behavior. Functionally, dysregulation of the 5-HT system may impair behavioral control, contributing to feather-pecking behavior [32]. Besides the 5-HT system, the levels and functions of other neurotransmitters, such as DA, also influence the occurrence of the feather-pecking behavior in goslings. This study revealed that DA exhibits higher levels in victims and lower levels in NFP goslings. DA plays a critical role in various physiological processes, including motor control, reward mechanisms, and emotional regulation [17,43]. Previous research has shown that the dopaminergic system is closely associated with feather-pecking behavior. Specifically, the central DA promotes the motivation to perform reward-related behaviors [44]. Through their respective functions, 5-HT and DA contribute to redirected feeding behaviors such as feather pecking. In conclusion, goslings with a high propensity for feather pecking exhibit reduced 5-HT levels.

The gut microbiota functions as a fully operational endocrine organ, producing, regulating, and releasing a variety of compounds that enter the host’s circulatory system. Through the functional “bridge” of the gut–brain axis, the gut microbiota influences organ function across the body and modulates the activity of the central nervous system. In this capacity, it exhibits a pivotal and distinctive role similar to that of an endocrine organ, extending its influence from the gut to the entire organism and participating in intricate physiological regulation. Using 16S rRNA amplicon sequencing, we analyzed microbial community dynamics and identified the predominant bacterial phyla in SFP, victim, and NFP goslings as *Firmicutes*, *Bacteroidetes*, and *Proteobacteria,* respectively. Among these, an increase in Firmicutes was observed in SFP goslings, consistent with previous findings of elevated Firmicutes levels in FP individuals. Interestingly, Borda-Molina et al. [45] reported that Firmicutes can produce or induce the host’s production of immunomodulatory factors, which are implicated in triggering inflammation, anxiety, and depressive states [46,47]. In addition, the abundance of *Faecalibaculum* spp., which prevents inflammatory responses and is associated with mood disorders, was reduced in the intestines of SFPs. In the gut flora of victims, an increase in the abundance of *Turicibacter* spp., which triggers an inflammatory response, was found [48,49]. In the intestines of NFP birds, an increased abundance of *Bacteroides* spp. and *Parabacteroides* spp. was observed. These bacterial genera are known to suppress specific immune responses [50] and have been implicated in neurodevelopmental processes [51]. Numerous studies have shown that *Bacteroides* are associated with depression, [52,53,54], which correlates with the results of this study, where *Bacteroides* abundance was reduced in SFPs and victims. FP is a maladaptive behavior that can be influenced by chronic stressors and the physical trauma associated with being pecked, which may negatively impact both the physiological and psychological state of the birds. This behavioral abnormality is closely linked to mood regulation, particularly through the serotonergic system. Reduced levels of 5-HT, a key neurotransmitter associated with mood regulation, have been identified as a significant neurobiological factor contributing to the development of depression [17]. Collectively, these findings suggest that both the gut microbiota composition and the 5-HT system may play pivotal roles in regulating feather-pecking behavior and depressive-like states in avian populations. Based on our research results, we propose the following targeted strategy: adding prebiotics or probiotics to the feed to restore the balance of the colonic microbiota, which may indirectly regulate serotonin metabolism.

## 5. Conclusions

In conclusion, through the analysis of behavioral characteristics, physiological indicators, and gut microbiota, our research explored the disparities in FP phenotypes among different gosling groups. Feather-pecked goslings in this study presented low feather scores and body weights, accompanied by poor antioxidant capabilities. The SFP goslings suffered from long-term stress and immunosuppression. Specifically, they had a low serum 5-HT content and a disrupted cecal microbiota. This was marked by an elevated abundance of *Bacteroides*, along with relatively high abundances of *Proteobacteria* and *Bilophila*. Our findings suggest that variations in cecal microbiota composition and 5-HT metabolism are pivotal in determining FP phenotypes. Exploring dietary interventions to enhance 5-HT synthesis represents a promising strategy to alleviate the low serum 5-HT levels observed in FP goslings.

## Figures and Tables

**Table 1:** Definition of gosling classification.

Behavior Pattern	Definition
Severe feather peckers (SFPs)	Goslings that are forcibly pulling, pecking, or plucking feathers from other birds and have a high frequency of feather pecking
Victims	Goslings with missing back feathers or even skin damage and scabs
Non-peckers (NFPs)	Goslings reared in low-density groups with no or very low frequency of pecking

**Table 2:** Feather scoring standard.

Score	Feathers	Part
5	Feathers in perfect condition	Chest, legs, back, tail and rump, wings, head, and neck
4	Feathers with damage, but no exposed skin areas	
3	Exposed area no larger than 3 × 3 cm	
2	Exposed area ranging from 3 × 3 cm to 5 × 5 cm	
1	Exposed area larger than 5 × 5 cm	

**Table 3:** Sequences of used primers for real-time PCR.

Gene	GenBank Accession	Primer Sequences (50−30)	Size (bp)
*SLC6A4*	XM_013196494.3	AGGCAACGAGCAATGAGA GCAGGGCAGATTTAGGGT	299
*HTR1A*	XM_048079550.2	CTGGAGATCATCGAGGTCC CAAGCGGTGTCACAAAAGG	257
*TPH-2*	XM_048065403.2	CTCACCCTAAACAGATCAA CAGTCACAGTCCACAAAAA	245
*GAPDH*	XM_067004670.1	CATGTTCGTGATGGGTGTG CTGGGATAATGTTCTGGGC	239

## Data Availability

All data generated or analyzed during this study are included in this published paper.
